# Genome microbiology at 20 years: who we are and where we are going

**DOI:** 10.1093/dnares/dsag016

**Published:** 2026-09-18

**Authors:** Shogo Ozaki, Satoru Watanabe, Taku Oshima

**Affiliations:** Department of Molecular Biology, Graduate School of Pharmaceutical Sciences, Kyushu University, Fukuoka 812-8582, Japan; Department of Bioscience, Tokyo University of Agriculture, Tokyo 156-8502, Japan; Department of Biotechnology, Toyama Prefectural University, Toyama 939-0398, Japan

**Keywords:** genome-based microbiology, microbial physiology, genomics and metagenomics

## Abstract

The International Symposium ‘*Microbial Growth and Behavior: From Bench to Nature’* was held as part of the annual meeting of the Society for Genome Microbiology, Japan. This session was designed to highlight challenges in modern microbiology: how to connect molecular insights obtained from laboratory model systems with the dynamic and complex responses of microbes in natural environments.

## 1. Introduction

For decades, classical microbiology performed under controlled culture conditions has elucidated fundamental principles of cell growth, stress signalling, chromosome dynamics, and regulatory networks, creating the foundation of modern molecular biology.^1–6^ However, natural environments expose microbes to fluctuating resources, spatial heterogeneity, multicellular interactions, and ecological pressures that are often difficult to recapitulate in the laboratory. Understanding how microbial cells behave under complex conditions requires bridging laboratory precision with ecological realism.

One of the reasons microbiology is expanding from a laboratory-based discipline towards a science that embraces natural and ecological complexity is the remarkable advance in genomics. High-throughput sequencing techniques have transformed phylogeny, enabling unprecedented resolution of evolutionary relationships across bacteria, archaea, and phages.^7–11^ Long-read sequencing technologies have also revealed epigenetic and epitranscriptomic regulatory mechanisms that influence diverse cellular processes in bacteria.^12–15^ Furthermore, sequence-based technologies such as ChIP-seq,^16–18^ Hi-C,^19–21^ and transposon mapping^22–25^ have enabled genome-wide analyses of protein–DNA/RNA interactions, chromosome organization, and gene essentiality. More recently, single-cell transcriptomic approaches, which have flourished in eukaryotic systems, are becoming feasible in prokaryotic organisms, offering new opportunities to investigate regulatory programmes at the level of individual cells and their cell-to-cell variations.^26–31^ Collectively, the insights emerging from genome-based microbiology have uncovered important regulatory layers that extend far beyond the genetic code.

Even more transformative has been the rise of metagenomic approaches, which allow researchers to directly sequence microbial communities and viral populations in natural environments.^32–34^ Metagenomics has led to the discovery of countless new species, novel metabolic pathways, and unexpected ecological interactions, as well as proteins whose biochemical properties are still unknown.^35–39^ These findings have also revealed extensive horizontal gene transfer and auxiliary metabolic genes carried by phages, which influence host evolution, physiology, and ecosystem functions.^40^ Collectively, these approaches have opened new perspectives on microbial evolution, horizontal gene flow, and the ecological strategies that microbes employ in diverse habitats, ranging from soil and freshwater to the human body and extreme environments.

Together, these genome-driven approaches have greatly expanded our view of microbial diversity and provided new insight into how microbial systems operate. They have also provided the essential context for integrating laboratory findings with ecological and host-associated biology. In this spirit, the symposium brought together researchers whose work exemplifies this integration spanning cytotoxic stress responses, morphological transitions, host-relevant social behaviours, and environmental sensory systems. Their presentations illustrated how molecular mechanisms discovered at the bench can explain microbial strategies in natural environments, and how environmental complexity, in turn, enriches and challenges our mechanistic understanding.

Dr. Setsu Kato from Hiroshima University presented state-of-the-art single-cell imaging approaches to dissect the cytotoxic effects of alcohol on *Escherichia coli*.^41,42^ Her work highlighted the remarkable cell-to-cell heterogeneity in subcellular dynamics and the timing of responses to cytotoxic stress. By resolving damage responses at the level of individual cells, she is uncovering how distinct stress-induced events are orchestrated within the microbial population.

Dr. Daisuke Shiomi from Rikkyo University discussed the physiology of *E. coli* L-forms, focusing on their unique cell-wall-deficient state.^43,44^ His talk emphasized the crucial role of chromosome dynamics in enabling L-forms to revert to the canonical rod-shaped lifestyle. This work provides important mechanistic insight into cellular plasticity and survival strategies under cell-wall-compromised conditions, which are frequently encountered by microbes in nature such as during host infection.

Dr. Urs Jenal from University of Basel used *Pseudomonas aeruginosa* as a model to illustrate how bacteria coordinate division of labour to optimize surface colonization.^45,46^ He first demonstrated in controlled laboratory settings that the second messengers c-di-GMP and cAMP govern cellular programmes to coordinate colonization efficiency. In particular, this coordination enables asymmetric daughter-cell fates following division: one daughter cell remains attached to the surface whereas the other disperses to explore new environments, thereby generating a division of labour that promotes efficient colonization. He then expanded these findings to human lung tissue infection models, revealing that the same regulatory principles operate in host environments, underscoring the ecological and clinical relevance of the mechanistic framework.

Dr. Gen Enomoto from Tokyo University of Agriculture presented elegant studies using cyanobacterial species to explore how these cells perceive and integrate different wavelengths of light to regulate motility and attachment.^47,48^ By sampling cyanobacteria from natural habitats and examining their behaviours under controlled laboratory conditions, his work provides a compelling example of how field-derived microbial diversity can illuminate adaptive strategies for life in fluctuating light environments.

## 2. Concluding remarks

A recurring message that now defines the Society for Genome Microbiology, Japan is that true progress in genome-based microbiology depends on iterative experimental cycles between environments and laboratories. Natural environments reveal the authentic selective pressures, ecological constraints, and evolutionary innovations that shape microbial genomes. Laboratory systems, in turn, provide the resolution necessary to mechanistically decode these discoveries at the molecular level. When researchers bring environmental or clinical isolates into the lab, test genome-derived hypotheses under controlled conditions, and then return to natural environments to validate and refine their findings, a powerful feedback loop emerges. This back-and-forth movement not only deepens our understanding of microbial physiology and evolution but also ensures that genome-driven discoveries remain grounded in reality. Genome technologies continue to advance and will be essential for the next generation of genome microbiology, one that plays an indispensable role within the broader field of general microbiology, where molecular precision and ecological relevance meet.
